# Migration of Leucocytes in Passive Hyperæmia

**Published:** 1878-09

**Authors:** Wm. T. Belfield


### MIGRATION OF LEUCOCYTES IN PASSIVE HYPERÆMIA.

By Wm. T. Belfield, M. D.

*Read at the National Microscopical Congress, August 15th.*

In March last I had occasion to superintend the *post mortem* examination of a case of pneumonia in which death had occurred on the tenth day of the disease. There was found consolidation of the entire left lung—gray hepatization in the lower lobe, red in the upper. Nothing peculiar was noticed in the other organs except general engorgement, especially marked in the kidneys. There had been, during our observation of the patient, not a solitary symptom of renal disease except the presence of albumen (about five per cent.) in the urine—a presence known to be common in pneumonia and attributed to mechanical congestion; for at least three features of pneumonia tend to the production of mechanical congestion: the decrease in breathing surface, the increase in the demand for oxygen made by the excessive tissue change, and the feebleness of the heart's contractions. Hence the albuminuria and the *post mortem* engorgement of the kidneys were regarded as legitimate results of the disease. A happy curiosity, however, led me to make a microscopic section of the kidney. I found the tubules rather smaller than usual, the intertubular spaces and capsules of the malpighian tufts much thickened by the presence within them of numerous small, round, finely granular cells $\frac{1}{2400}$ to $\frac{1}{3000}$ inch in diameter. These cells had every appearance of white blood corpuscles and were so considered by Dr. Danforth and others. By way of explanation it was presumed that the retardation of the blood-current due to the causes previously mentioned, had afforded the colorless corpuscles an opportunity to exhibit their amœboid movements, and that the opportunity had been improved. That this was not an inflammatory process was proved by the absence from the clinical history of all the recognized symptoms of renal inflammation, and by the absence from the urine of the exudation cylinders or tube-casts.

Nor were the microscopic appearances of the organ those of inflammation.

Within six weeks I had an opportunity of examining the kidneys of two other patients dead from pneumonia without previous history of renal disease. In each case the urine contained a small quantity of albumen but no casts, and in each instance colorless corpuscles were found in abundance in the intertubular tissue and in the malpighian capsules. At a meeting of the West Chicago Medical Society, held June 10th, I exhibited a section from one of these kidneys and another from a normal kidney, demonstrating to the satisfaction of those present the existence, within the connective tissue of the former organ, of the small round cells above described. At that time I had sought unsuccessfully for literature or a reference to literature on this subject. However, deeming the fact, if it were a fact, of the migration of leucocytes in passive hyperæmia a very important one pathologically, I determined to investigate the case, and for that purpose instituted the proceedings about to be narrated.

On June 19th I curarized a frog, cut down on the femoral vein, made compression by means of a rubber band and a piece of cork (which was readily done without compressing the artery, as in the frog the vein and artery lie on opposite sides of the femur) and stretched the web of the corresponding foot on the stage of the microscope. I employed a Hartnack No. 4 objective, and a Gundlach C. periscopic eye-piece. By watching the blood movements I easily regulated the pressure so as to retard more or less completely the onward movement, avoiding complete stagnation. After considerable pressure had been exerted, as was shown by accumulation of blood corpuscles, distension of the veins, and retardation of the current, the field was carefully watched for nine hours. During this time no leucocytes were actually observed to leave the vessels, yet several were seen just external to the walls, having apparently escaped unnoticed during the shifting of the stage. For the next ten hours the field was not observed with sufficient care and frequency to warrant any assertion of migration. At the end of this period, however, that is nineteen hours after compression was made, an almost continuous observation of the field was begun. From the 19th to the 36th hour leucocytes were observed to leave the vessel in considerable numbers, the shortest time of exit observed being twenty minutes—the average, one to two hours. The method of locomotion did not of course, differ from that exhibited in inflammation, though at no time did I observe the excessive change of form and protrusion of long processes figured in the books. There was frequently a flattening of the leucocyte against the wall; then the appearance of a bud external to the wall; then the gradual enlargement of this bud and shrinkage of the intra-vascular portion, the part piercing the wall being apparently a tunnel through which the rest of the body passed. Often the locomotion was continued after the leucocyte had become wholly extra-vascular, so that it traveled several times its own diameter from the place of exit. It was noticed, too, that other corpuscles were prone to pass out at the particular point of previous emigration, so that sometimes several would be crowded together along the vascular wall, and an hour later would be in close proximity external to the vessel opposite the same point. This phenomenon occurred usually not in the minutest capillaries, but rather in the large capillaries and small veins, ranging from $\frac{1}{1500}$ to $\frac{1}{600}$ inch in diameter. Nor were the passages always made where the current was slowest, nor where the vessel gave evidence of greatest engorgement, as emigration from a relatively rapid current, but sparsely supplied with corpuscles, was not infrequent.

Before I had watched the process a great while, I became aware that the colorless corpuscles were not the only bodies exhibiting amœboid movements. I observed that certain red ones, without nuclei, of circular shape and small size ($\frac{1}{2500}$ inch in diameter), performed the same movements with as great celerity as did the white. (The reader is reminded that in the frog the perfect red corpuscle is of elliptical shape is $\frac{1}{1200}$ inch in its longer diameter, and has a distinct nucleus; while the white globule is, when at rest, of circular shape, its diameter being only about $\frac{1}{2500}$ inch.) There could be no possibility of confounding these small red ones with the white; for although in size, shape and movements they were identical the red color of some was unmistakeable. So extensive was the locomotion of the red ones that at the 36th hour of the experiment there were numerous red patches in the field, which looked almost like hæmorrhages. That they were not hæmorrhages I knew, first because there were no large oval corpuscles, the red globules proper, but only the small circular ones; second, because they were fixed in the tissue, not floating to and fro in blood serum; third, because I had seen many of them pass slowly through the vascular walls.

These phenomena were witnessed not by myself alone. Dr. Bridge observed the emigration of several corpuscles; Dr. Danforth while unable to watch the process for a considerable time, was convinced that emigration actually occurred; Miss Meigler observed numerous red as well as white cells leave the vessels.

Having established the fact of the locomotion, therefore, it remains to prove the dependence of that locomotion upon mechanical congestion rather than upon active hyperæmia. That congestion existed was shown not only by the retardation of the blood current and dilatation of the vessels, but also by œdema of the web, which became evident within twenty-four hours. That the congestion was not "active" was established by two facts—first, the absence of all the phenomena of inflammation, other than the amœboid movements—i. e., the primary acceleration of the blood current, the subsequent retardation with irregular contractions of the vascular walls, etc.; second that the discontinuance of the pressure on the vein was at once followed by complete restoration of the circulation in the web, whose irregularities therefore, were dependent wholly on a mechanical impediment, and not upon any "nutritive irritation," nor vascular spasm.

This experiment has been repeated twice since the above date. In both instances the same phenomena occurred; in one, emigration began within three hours after compression was made. In this instance the pressure excited, exceeded somewhat that made in the first case.

Now the value of these facts depends upon one's ideas of pathogenesis. If he believes, with Billroth, that connective tissue is developed solely from migrated blood corpuscles, he has a key at once to the connective tissue hyperplasia of the skin and venous walls, which accompanies a varicose condition of the veins. For the mechanical congestion necessarily present must result in the emigration of blood corpuscles into the surrounding tissues, and these are developed, says Billroth, first into spindle-cells, and finally into complete connective tissue corpuscles, causing the familiar thickening of the skin and vascular walls. In support of this view is the fact that the increase in thickness in the wall of a varicose vein is due to hyperplasia, not of the muscular elements, but of the connective tissue bundles interposed among those elements, and of the outer coat of the vessel, which is composed wholly of connective tissue. So, too, the enlargement of the spleen which usually follows portal obstruction as in cirrhosis of the liver, and the thickening of the superior hæmorrhoidal veins—" hæmorrhoids "—from the same and other conditions, are to be referred, in part at least, to the development of leucocytes which have wandered from the vessels during the mechanical congestion. Perhaps, too, the areolar hyperplasia so often found with displacements of the uterus is due to the venous congestion which usually exists in that condition.

Even the conservatives, like Stricker and Rindfleisch, while insisting on the proliferation of pre-existing cells as the more important source of connective tissue hyperplasia, admit the strong probability that a considerable part of it is due to the development of the wandering cells or colorless corpuscles first into spindle-cells, then into fibrillated tissue. Certain it is that in the repair of wounds, at least, they play a prominent part in the formation of cicatricial tissue.

The fact of emigration without any evidence of that " nutritive irritation " of tissue which Virchow presumes in active hyperæmia would seem to favor Hering's view that the exit of corpuscles is a passive rather than an active movement, due to their glutinosity, to increased blood pressure and diminished blood velocity—in short, a simple filtration of colloid substances.

Then again the behavior of the small red corpuscles is interesting as exhibiting their close relation with the white, and as furnishing another link in the chain of circumstantial evidence that the red corpuscles are transformed white ones. Such has for some time been the prevalent opinion, though never completely demonstrated. The fact that these large ones were but little, if any, larger than the white, that they were of circular shape, were devoid of nuclei and possessed the power of amœboid motion, proves their close connection with the colorless ones; while the presence of hæmo-globin, as indicated by their color, testifies to their ability to perform at least one important function of the fully developed red ones—the transportation of oxygen.

In examining further the literature of this subject, I find in Wagner's Pathology (p. 186) Charlton Bastian cited as authority for the statement published in the *British Medical Journal* for 1868, that "red globules as well as white leave the vessels in venous stases, scorbutus, etc., by means of amœboid movements." Not having had access to the files of that journal I know nothing of his observations. The red globules mentioned are probably the small ones only; certainly I failed to observe the locomotion of any large oval discs.

